# Supplementary material for: Direct measurements of the colloidal Debye force
Source: Nat Commun. 2023 Jun 29;14:3838. doi: 10.1038/s41467-023-39561-8 (PMC10307906; doi:10.1038/s41467-023-39561-8)
Supplement: Supplementary file 1 — Supplementary Information [file 41467_2023_39561_MOESM1_ESM.pdf]

Supplementary Information

Direct measurements of the colloidal Debye force

Hyang Mi Lee [1,†], Yong Woo Kim[1,†], Eun Min Go[2,†,‡], Chetan Revadekar[1], Kyu Hwan Choi[1,#], Yumi Cho[2], Sang Kyu Kwak[3,*], and Bum Jun Park[1,*]

[1]Department of Chemical Engineering (BK21 FOUR Integrated Engineering Program), Kyung Hee University, Yongin, Gyeonggi-do 17104, South Korea

[2]School of Energy and Chemical Engineering, Ulsan National Institute of Science and Technology, Ulsan 44919, South Korea

[3]Department of Chemical and Biological Engineering, Korea University, Seoul 02841, South Korea

[†]These authors equally contributed to this work.

[‡]Current address: Corning Technology Center Korea, Corning Precision Materials Co., Ltd., 212 Tangjeong-ro, Asan, Chungcheongnam-do, 31454, South Korea

[#]Current address: Department of Chemical Engineering, University of California Santa Barbara, Santa Barbara, CA 93106, USA

[*]Corresponding authors: (email) skkwak@korea.ac.kr; bjpark@khu.ac.kr

Supplementary Table 1. Properties of PS particles.

	Sulfate-PS (SPS)	Amino-PS (APS)	Carboxyl-PS (CPS)
Diameter, d (μm)	2.96 ± 0.05	2.79 ± 0.11	3.16 ± 0.07
ζ-potential, ψ (mV)	–57.5 ± 2.2	–69.5 ± 2.4	–65.6 ± 3.2

Supplementary Note 1: Optical laser tweezer setup.

To set up the optical laser tweezers, we used an inverted microscope (Ti-U, Nikon, Japan) along with a 10W CW Nd:YAG laser with a 1064 nm wavelength.[1-3] The laser beam passed through an acousto-optic deflector (AOD, Opto-electric DTSXY-400-1064 2D, AA Opto Electronic, USA) before entering a water immersion objective (CFI Plan Apochromat VC 60×, Nikon, Japan) with a numerical aperture of NA = 1.2 and a working distance of ~300 μm. By focusing the beam on a specific focal plane, an optical trap was generated. The x and y positions of the trap were adjusted by diffracting the laser beam through the AOD, which was operated with LabVIEW software. Multiple optical traps were generated by time-sharing a single beam using the AOD, and their trap stiffnesses were equalized by implementing a consistent pause time at each discrete trap position.[3] While each trap stiffness increased with laser power, it decreased with the number of time-shared traps generated. To measure the pair interaction force, we typically generated a total of 10 optical traps, each receiving an allocated laser power of ~7 mW, making the total measured laser power ~70 mW. The laser power was directly measured using an optical power meter (PM100D, Thorlabs, USA) positioned above the objective.

Supplementary Note 2: Drag calibration.

The trap stiffness κ_t was measured by using the drag calibration method, which involved subjecting a trapped particle to drag force by moving a motorized microscope stage (SCAN^{plus} 130×85, Märzhäuser Weltzlar GmbH & Co. KG, Germany) at varying constant velocities between u = 5– 70 μm·s^{-1}.[3-5] The displacement of the particle Δx from its equilibrium position was caused by the Stokes drag force $F_{\mathrm{Stokes}} = 3\pi d\eta_{\mathrm{w}}u$, where η_{w} is the water viscosity. A plot of F_{Stokes} versus Δx was used to obtain the trap stiffness κ_t via linear regression. This drag calibration was performed at several AOD setting values corresponding to particle positions along the x-direction, where the pair interaction measurements were conducted. The measured κ_t values did not vary significantly with the AOD setting values, as shown in the inset of Supplementary Fig. 1, and therefore, their mean value of $\langle\kappa_t\rangle = 3.91 \pm 0.07$ pN·μm^{-1} was used in this study.

To validate the measured κ_t values, we calculated the optical trapping force numerically using the ray optics approximation.[6] We refer the readers to our previous work for the detailed calculation method.[5, 7] Under the experimental conditions with laser power P = 7 mW, water and PS refractive indices of $n_1 = 1.326$ and $n_2 = 1.57$, NA = 1.2, and the particle diameter d = 2.96 μm, the calculated trapping force or gradient force F_{trap} was plotted in Supplementary Fig. 1. The linear regression of the linear regime (light orange region) resulted in $\kappa_t = 3.85 \pm 0.06$ pN·μm^{-1}, which showed excellent agreement with the experimental value.

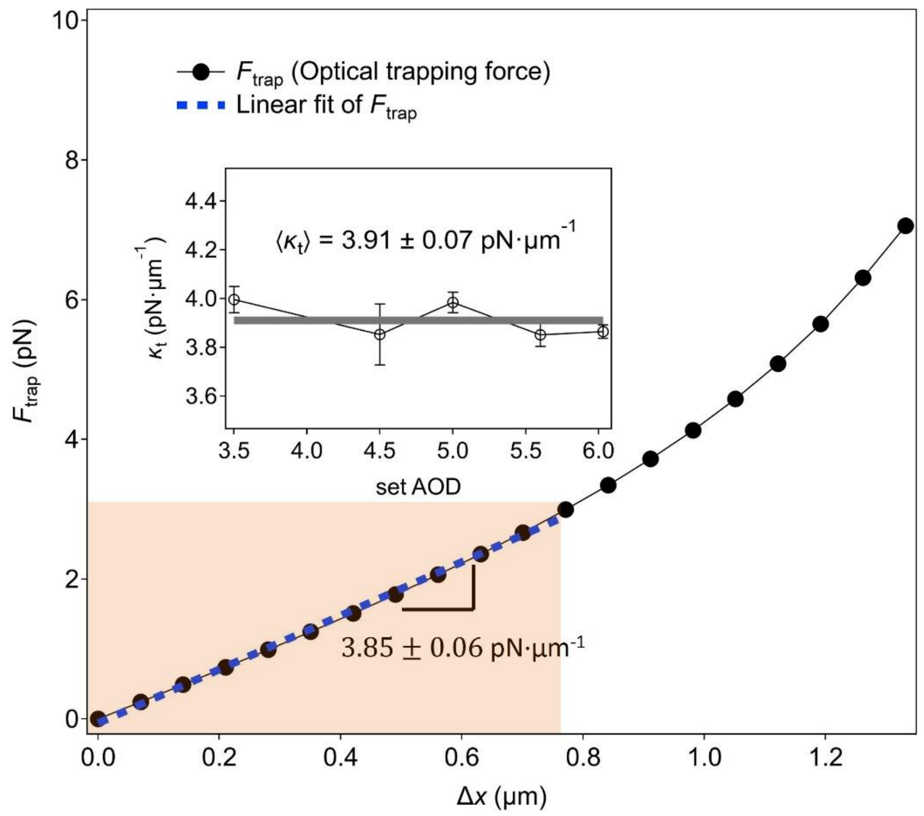

Supplementary Fig. 1. Optical trap calibration. Numerical calculations of the optical trapping force F_{trap} as a function of lateral displacement Δx at a laser power of P = 7 mW. The linear fit in the light orange region estimates the trap stiffness about $\kappa_{\mathrm{t}} = 3.85 \pm 0.06$ pN·µm^{-1}. The inset shows the results of drag calibration experiments conducted at various set values of AOD, which correspond to the x-positions of the optical trap used in the pair interaction measurements. Each data point in the inset plot indicates the mean value of three-independent runs and the corresponding standard deviation of the drag calibration.

Supplementary Note 3: Image analysis.

A microscopic movie was recorded using a charge-coupled device (CCD) camera (Hitachi, KP-M1AN, Japan) and/or a complementary metal-oxide-semiconductor (CMOS) camera (CS505CU, Thorlabs, USA), installed in the Ti-U inverted microscope, at a rate of 30 frames per second (fps). The exposure time and magnification were 16.7 ms and 4.41 pixel·μm^{-1} for the CCD camera and 29.998 ms and 17.55 pixel·μm^{-1} for the CMOS camera, respectively. The recorded movie was saved as a sequence of microscopic images using the ImageJ software.[8] Particle positions were analyzed using a standard particle tracking routine[9] implemented with MATLAB.[10] Notably, for the pair interaction force measurements reported in Fig. 2d, cases where two particles did not contact each other were only considered. As a result, the possibility of any overlapping effects of the two particles causing artifacts during the image analysis process could be minimized.

Supplementary Note 4: General formulas for interactions between molecules.[11, 12]

When two permanent point dipoles μ_1 and μ_2 are fixed at a mutual orientation angle of θ and separated by a distance r in a medium, their dipole-dipole (D–D) potential energy can be expressed as $U_{\mathrm{D-D,fixed}} = \frac{\mu_1\mu_2 f(\theta)}{4\pi\varepsilon_0\varepsilon_\mathrm{r}} r^{-3}$, where ε_0 is the vacuum permittivity, ε_r is the dielectric constant of the medium, and $f(\theta) = 1 - 3\cos^2\theta$. The r^{-3} dependence arises from the field strength of the two point dipoles (r^{-1} dependence) and the magnitude of each dipole decreasing as r increases (r^{-2} dependence). For two freely rotatable dipoles, their relative orientation is constrained by the interaction strength (r^{-3} dependence). The Keesom interaction, which is the first contribution to the van der Waals (vdW) interaction, is always attractive and can be described by combining the Boltzmann equation (r^{-3} dependence) with $U_{\mathrm{D-D,fixed}}$: $U_{\mathrm{D-D,free}} = -\frac{2}{3k_\mathrm{B}T}\frac{\mu_1^2\mu_2^2}{(4\pi\varepsilon_0\varepsilon_\mathrm{r})^2} r^{-6}$, where k_B is the Boltzmann constant and T is the temperature.

When nonpolar molecules are exposed to an electric field, their electronic distribution and nuclear positions are distorted, inducing a temporary dipole moment. For moderate field strengths E, the magnitude of the induced dipole moment μ^* is linearly proportional to E and can be described by $\mu^* = \alpha E$, where α represents the molecular polarizability. The polarizability volume α' can be expressed as $\alpha' = \frac{\alpha}{4\pi\varepsilon_0\varepsilon_\mathrm{r}} = \frac{\mu^*}{4\pi\varepsilon_0\varepsilon_\mathrm{r}E}$. A permanent dipole μ_1 can induce a dipole moment μ_2^* in a nonpolar, polarizable molecule. The interaction between the dipole and the induced dipole (D–I) is attractive and is referred to as the Debye interaction, which is the second contribution to the vdW interaction. The Debye interaction potential is given by $U_\mathrm{D-I} = -\frac{1}{2}\alpha_2 E^2$, where $E = \frac{\mu_1(1+3\cos^2\theta)^{\frac{1}{2}}}{4\pi\varepsilon_0\varepsilon_\mathrm{r}r^3}$ is the electric field generated by μ_1. When the Debye interaction is not strong enough to mutually orient the molecules, the angle average of $\cos^2\theta = \frac{1}{3}$ is used to describe $U_\mathrm{D-I}$, which is given by $U_\mathrm{D-I} = -\frac{\mu_1^2\alpha_2}{(4\pi\varepsilon_0\varepsilon_\mathrm{r})^2}r^{-6} = -\frac{\mu_1^2\alpha_2'}{4\pi\varepsilon_0\varepsilon_\mathrm{r}}r^{-6}$. The inverse sixth power arises from the r^{-3} dependence of the magnitude of the induced dipole, which is weighted by the r^{-3} dependence of the interaction between the dipole and the induced dipole. The corresponding D–I interaction force is given by $F_\mathrm{D-I} = -\frac{\mathrm{d}U_\mathrm{D-I}}{\mathrm{d}r} = F_0\left(\frac{d}{r}\right)^7$, where $F_0 = -\frac{3\mu_1^2\alpha_2'}{2\pi\varepsilon_0\varepsilon_\mathrm{r}d^7}$. When the two molecules are mutually oriented with $\theta = 0°$ and thus $\cos^2\theta = 1$, the attraction is doubled, i.e., $U_\mathrm{D-I}(\theta = 0°) = -\frac{\mu_1^2\alpha_2'}{2\pi\varepsilon_0\varepsilon_\mathrm{r}}r^{-6}$.

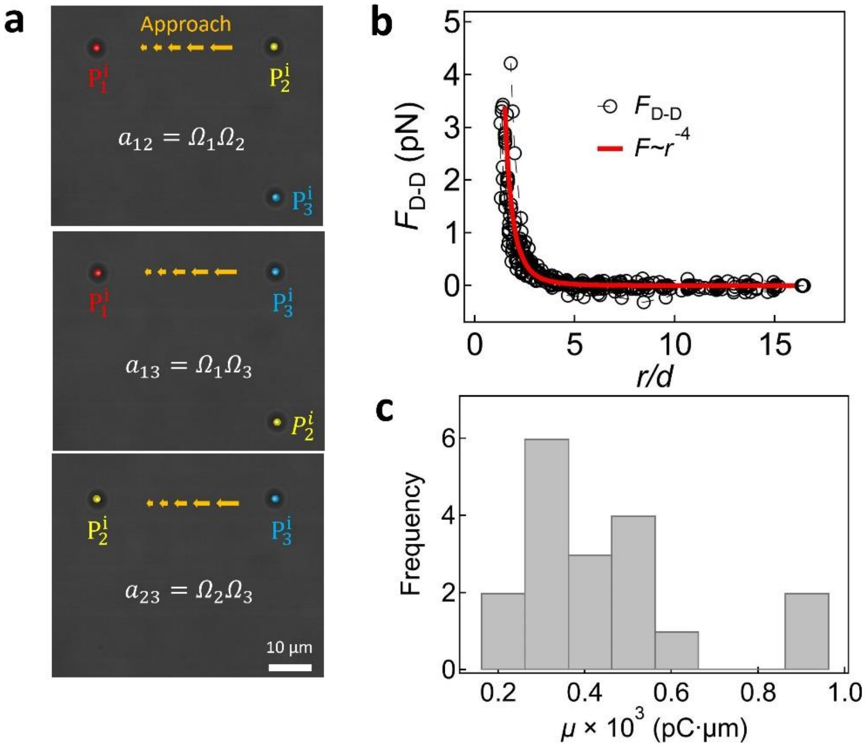

Supplementary Fig. 2. Determination of permanent dipole strength for the interface-trapped particles. a, Microscope images showing the pair interaction forces between SPS particles at a planar oil–water interface using the self-potential method. The aqueous subphase contains 10 mM NaCl. **b,** The measured force profiles for 18 pairs. The red solid line indicates the mean force profile. **c,** Histogram of the obtained μ values for the 18 particles.

Supplementary Note 5: Dipole strength of an interface-trapped particle.

When a charged colloidal particle is attached to an oil–water interface, an electric dipole can be formed due to the asymmetric surface charge dissociation across the interface.[13-15] The D–D interaction potential between two particles at the interface and the corresponding force are given by $U_{\mathrm{D-D}} = \frac{\mu_i \mu_j}{8\pi\varepsilon_0\varepsilon_{\mathrm{oil}} r^3}$ and $F_{\mathrm{D-D}} = -\frac{\mathrm{d}U_{\mathrm{D-D}}}{\mathrm{d}r} = \frac{3\mu_i\mu_j}{8\pi\varepsilon_0\varepsilon_{\mathrm{oil}} d^4}\left(\frac{d}{r}\right)^4$, respectively, assuming that each dipole is perpendicular to the interface.[16] Note that this expression differs by a factor of 0.5 and the use of the oil dielectric constant $\varepsilon_{\mathrm{oil}}$ compared to the standard version of the D–D interaction. These differences reflect that when two point dipoles, μ_i and μ_j, located at the interface are aligned

perpendicularly to the interface, the resulting D–D interaction is mainly originated by the electric field overlapping in the oil phase, rather than in the aqueous phase.

The dipole strength of the interface-trapped particles could be determined by the self-potential method.[13] The flow cell for the force measurement using optical laser tweezers was prepared using a previously reported method.[13] As shown in Supplementary Fig. 2a, three particles ($\mathrm{P_1^i}, \mathrm{P_2^i}, \mathrm{P_3^i}$) were optically trapped at the oil-water interface containing 10 mM NaCl in the aqueous subphase. After the pair interaction force (dipole-dipole interaction force, $F_{\mathrm{D-D}}$) of each pair (i.e., $\mathrm{P_1^i} - \mathrm{P_2^i}, \mathrm{P_1^i} - \mathrm{P_3^i}, \mathrm{P_2^i} - \mathrm{P_3^i}$) was measured, each force profile was fitted to obtain the interaction magnitude a_{ij} (a_{12}, a_{13}, a_{23}) using $F_{\mathrm{D-D}} = F_{ij} = -\frac{\mathrm{d}U_{ij}}{\mathrm{d}r_{ij}} = \frac{3a_{ij}k_{\mathrm{B}}T}{d}\left(\frac{d}{r_{ij}}\right)^4$. The interaction potential is $\frac{U_{ij}}{k_{\mathrm{B}}T} = a_{ij}\left(\frac{d}{r_{ij}}\right)^3 = \Omega_i\Omega_j\left(\frac{d}{r_{ij}}\right)^3$, where k_{B} is the Boltzmann constant, T is the temperature, d is the particle diameter, r_{ij} is the center-to-center separation between the two particles, and Ω is the self-potential defined as that carried by each individual particle. The three unknown Ω values could be calculated from the three independent equations of $a_{ij} = \Omega_i\Omega_j$ $(i, j = 1,2,3\ \&\ i \neq j)$; for example, $\Omega_1 = \sqrt{\frac{a_{12}a_{13}}{a_{23}}}$. The value of Ω is proportional to dipole strength μ, which accounts for the strong electrostatic repulsion between the particles at the interfaces, given by $\frac{U_{ij}}{k_{\mathrm{B}}T} = \Omega_i\Omega_j\left(\frac{d}{r_{ij}}\right)^3 = \frac{\mu_i\mu_j}{8\pi\varepsilon_0\varepsilon_{\mathrm{oil}}d^3k_{\mathrm{B}}T}\left(\frac{d}{r_{ij}}\right)^3$ and $\mu = \Omega\sqrt{8\pi\varepsilon_0\varepsilon_{\mathrm{oil}}d^3k_{\mathrm{B}}T}$. As shown in Supplementary Fig. 2b, the interaction forces for 18 pairs were measured, and the mean force profile (red solid line) for the measured forces was consistent with the dipole-dipole interaction model ($F_{\mathrm{D-D}} \sim r^{-4}$). The histogram in Supplementary Fig. 2c represented the obtained μ values for the 18 particles, and their mean value was $\langle\mu_1\rangle \times 10^4 = 4.5 \pm 2.0$ pC·μm.

Supplementary Note 6: Pair interactions in 10 mM NaCl water.

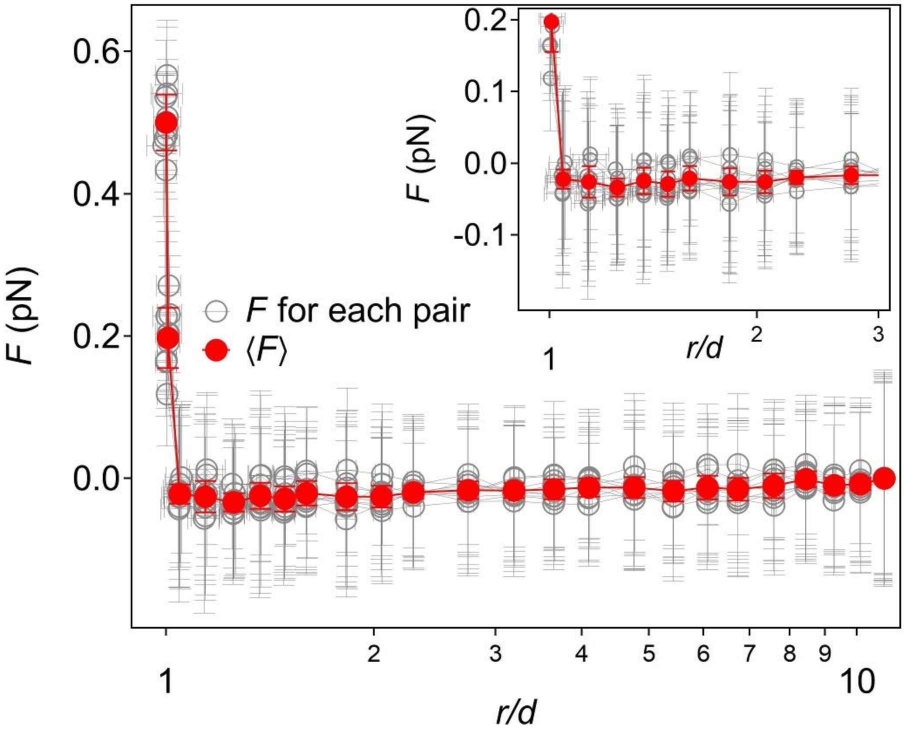

Supplementary Fig. 3. Pair interaction forces for nine different pairs suspended in 10 mM NaCl water. The grey circles represent the force profile for each pair, with the z-axis error bars indicating thermal fluctuations observed while holding the particles with optical tweezers. The red circles represent the average force profile over the nine pairs, with the error bars indicating the corresponding standard deviation. The x-axis is shown in log scale. The inset provides a magnified view of the force profile near separations.

Pair interaction forces were measured for SPS particles in 10 mM NaCl water to compare them with the D–I forces. To measure the interaction force between two particles in the 10 mM NaCl water-only condition, one particle P_1 was fixed with a stationary trap, and the other particle P_2 approached the P_1 particle with a translational trap stepwise. The displacements Δx of the stationary particle from its equilibrium position were measured as a function of the particle separation r, and Δx was converted to the pair interaction force using $F(r) = \kappa_t \Delta x(r)$.[14] A total nine different pairs were measured, and a nonsignificant negative force was detected near

separations, as shown in Supplementary Fig. 3. Note that the positive force in Supplementary Fig. 3 represents that the optical traps holding the two particles could not push more closely together, and the P_1 particle was pushed backward in the approach direction of the P_2. In addition, after the two particles approached each other closely, optical laser tweezers could readily separate them without the presence of measurable forces. The result of this force measurement in 10 mM NaCl water was consistent with the dimer formation probability in the same fluid condition, in which only six of 256 pairs formed aggregate dimers ($P_f \approx 2\%$), as in Fig. 3c.

The Derjauin-Landau-Verwey-Overbeek (DLVO) interaction theory provides a framework for describing colloidal interactions in an aqueous phase.[12, 17] For two spherical colloids with an equal diameter d, the vdW interaction can be expressed as $U_{\mathrm{vdW}} = -\frac{A_{\mathrm{H}} d}{24(r-d)}$, where $A_{\mathrm{H}} = 1.4 \times 10^{-20}$ J is the Hamaker constant between two PS particles interacting across water.[12] The double layer interaction between two colloidal spheres is given by $U_{\mathrm{el}} = 32 \times 10^3 \pi d k_{\mathrm{B}} T I N_{\mathrm{A}} \kappa^{-2} \Upsilon_0^2 \exp\left(-\kappa(r-d)\right)$ for $\kappa\left(\frac{d}{2}\right) > 10$, where $I = 10$ mM is the ionic strength, N_{A} is Avogadro's number, $\Upsilon_0 = \tanh\frac{e\psi}{4k_{\mathrm{B}}T}$ is the Gouy-Chapman parameter, $\psi = -57.5$ mV is the particle zeta-potential, e is the elementary charge, and $\kappa = \sqrt{\frac{2000 e^2 I N_{\mathrm{A}}}{\varepsilon_{\mathrm{w}} \varepsilon_0 k_{\mathrm{B}} T}}$ is the inverse Debye screening length.[17, 18] Note that the U_{el} equation agrees well with the numerical solution of the non-linear Poisson-Boltzmann equation, even at small interparticle separations ($\kappa(r-d) < 1$).[19] The corresponding force can be calculated numerically as the derivative of the potential energy with respect to interparticle separation, i.e., $F = -\frac{\mathrm{d}U}{\mathrm{d}r}$. The DLVO interaction forces between two particles, where one particle is attached to the oil–water interface and the other is immersed in water (Fig. 2d and 2e), were also estimated using the above equations.

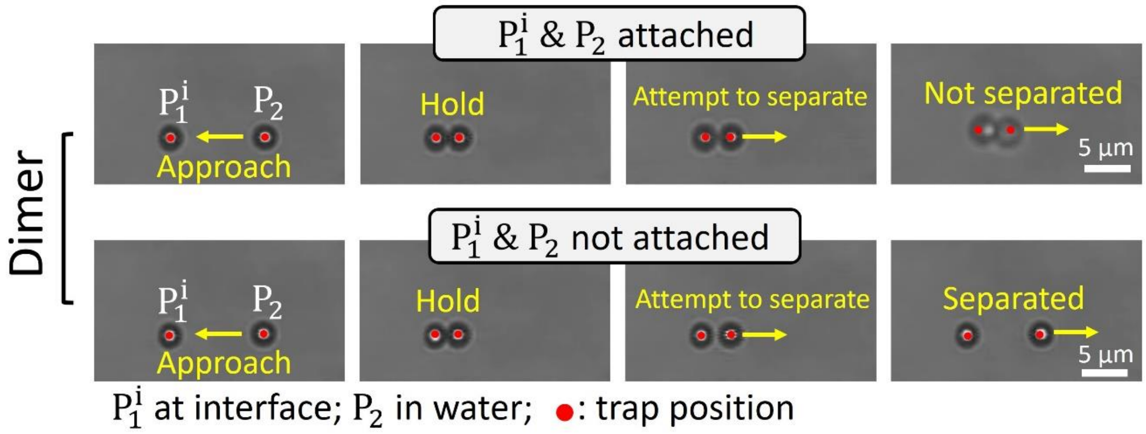

Supplementary Fig. 4. Evaluation procedure of dimer formation. P_1^i are P_2 are at the oil-water interface and in water, respectively (Supplementary Movies 3 and 4). The water phase contained 10 mM NaCl.

Supplementary Note 7: CGMD simulations.

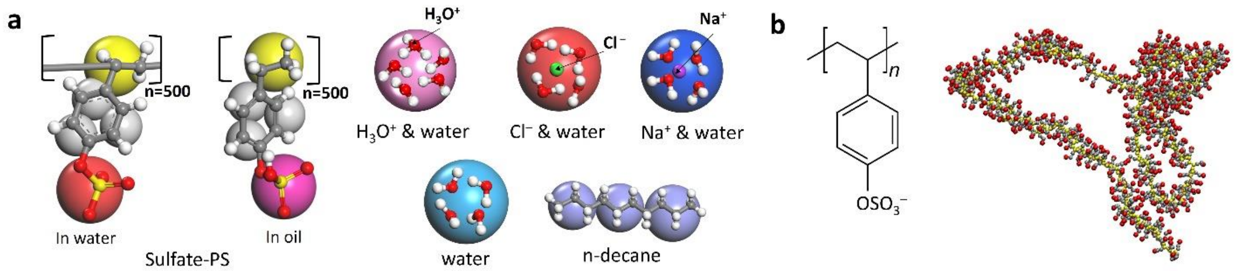

Supplementary Fig. 5. CGMD models for SPS and solvents. a, White, grey, red, yellow, and green represent hydrogen, carbon, oxygen, sulfur, and chlorine atoms, respectively. Note that charges were introduced to the beads, which OSO_3^-, $H3O^+$, Cl^- and Na^+. **b,** CG models for SPS (*n* = 500).

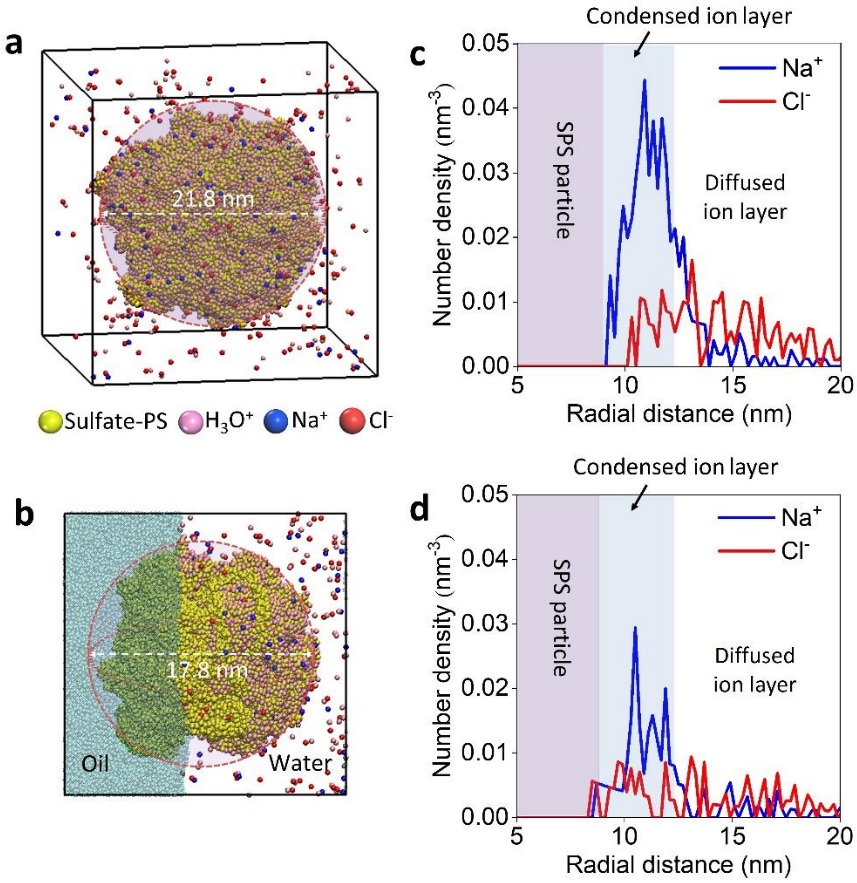

Supplementary Fig. 6. CGMD simulation of a single SPS in water and at the interface. a,b, Example configurations of the SPS particle and ions in water (**a**) and at the interface (**b**). **c,d,** Ion number density along the radial distance from the center of particle in water (**c**) and at the interface (**d**). Water molecules were omitted for clarity.

The ion distributions around a single SPS particle were analyzed in both conditions, in water (Supplementary Fig. 6a,c) and at the oil-water interface (Supplementary Fig. 6b,d). In both cases, a condensed layer of Na^+ ions was formed near the SPS surface exposed to the water phase, and a layer of diffusive ions was observed beyond it (Supplementary Fig. 6c,d).

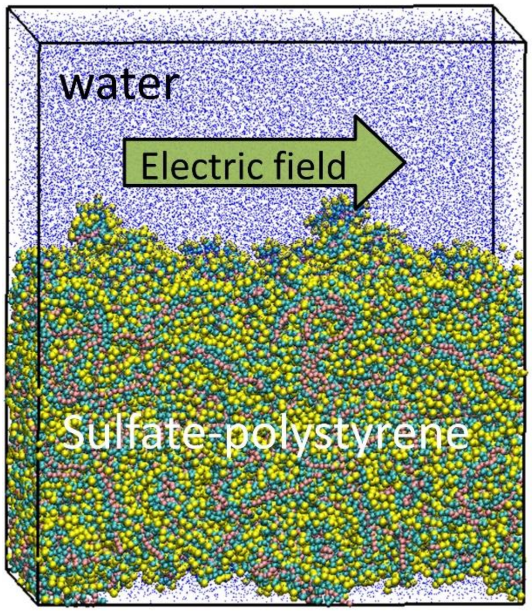

Supplementary Fig. 7. Simulation model system for calculating the ζ-potential of an SPS surface.

Previous work was followed for the ζ-potential calculation.[20] The thickness of a simulated SPS layer was 12 nm and an electric field of E_z = 0.01 V/nm was applied (Supplementary Fig. 7). The ζ-potential was obtained from the Helmholtz-Smoluchowski equation $\zeta = -\frac{\mu_z \eta}{\varepsilon_r \varepsilon_0}$, where η, ε_r, and ε_0 were the dynamic viscosity of a fluid, the relative permittivity, and the vacuum permittivity, respectively. The bulk fluid mobility relative to the SPS surface was given by $\mu_z = \frac{v_z}{E_z}$, where v_z was the fluid velocity. The obtained ζ-potential from the simulation was –48.2 mV, which was slightly lower than the experimental value (–57.5 mV). We used this SPS surface for further CGMD simulations.

The CGMD simulation aimed to confirm several aspects: firstly, the dipole formation due to the asymmetric ion distribution around the P_1^i particle attached to the interface; secondly, the induced ion rearrangement around the P_2 particle dispersed in water by the dipole of the P_1^i particle;

thirdly, the existence of relatively long-range attraction (i.e., D–I attraction), and fourthly, the confirmation of the difference in behavior between two particles in water-only and decane/water environments. It is worth noting that scaling up the simulation results to the experimental scale may increase the relative contribution of D–I attraction compared to vdW attraction, since the D–I potential is proportional to $\sim d^4$ and the vdW potential is proportional to $\sim d$, where d is the particle diameter. Nonetheless, interpreting the above items based on the results of the CGMD simulation would be reasonable.

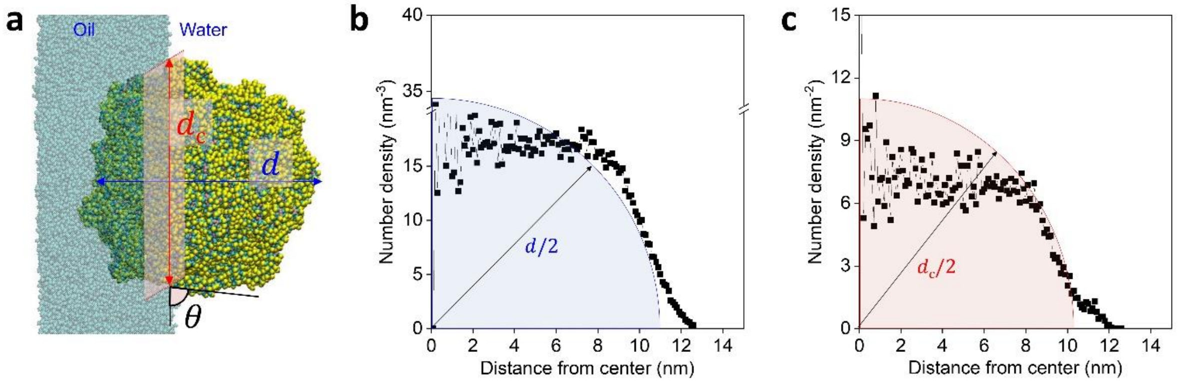

Supplementary Fig. 8. Estimation of the three-phase contact angle θ of a simulated SPS nanoparticle at the oil–water interface. a, Molecular configurations of the SPS particle at the interface with the particle diameter d and contact line diameter d_c, depicted in blue and red lines, respectively. Water molecules were not shown for clarity. **b,** Number density of the CG SPS beads in the water phase as a function of the radial distance from the particle center. **c,** Number density of the CG SPS beads of the cross-sectional area by the oil–water interface as a function of the radial distance from the cross-section center.

The three-phase contact angle θ of a simulated nanoparticle at the oil–water interface was calculated using $\sin\theta = d_c/d$, where d and d_c are the particle diameter and the dimeter of the cross-sectional area of the particle at the interface, respectively (Supplementary Fig. 8a). To

estimate its effective diameter, the number densities of the coarse-grained (CG) SPS beads were analyzed along the radial direction from the particle center for d (Supplementary Fig. 8b) and from the cross-section center for d_c (Supplementary Fig. 8c). The effective diameter was determined at the second inflection point of the number density. It was found that $d_c \approx 20.4$ nm and $d \approx 21.6$ nm, resulting in $\theta \approx 70.8°$.

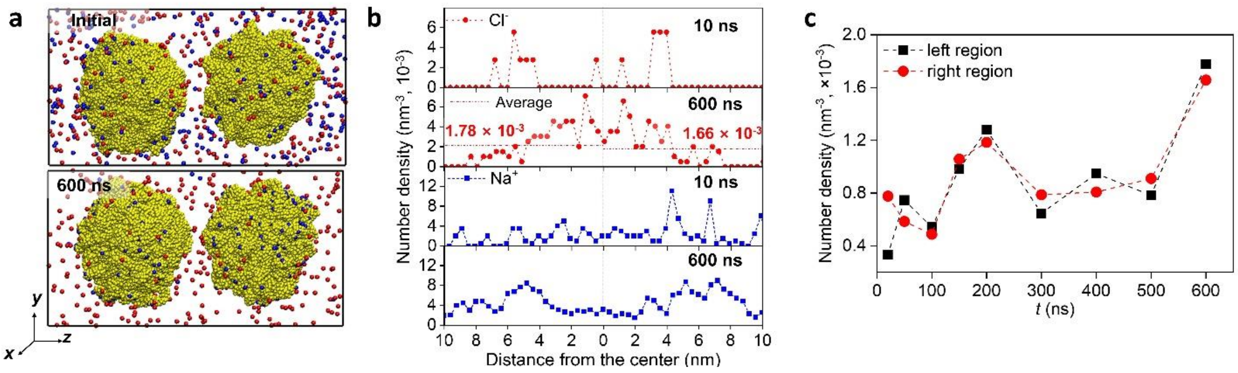

Supplementary Fig. 9. CGMD simulation in water when two particles are initially separated with a finite surface-to-surface distance. a, Initial and final configurations of two water-immersed SPS particles. Yellow, red, and blue represent SPS, Na^{+}, and Cl^{-}, respectively. Water molecules were omitted for clarity. **b,** Number density of Cl^{-} and Na^{+} between the two particles over the simulation time. The black dashed line represents the center of the two particles. **c**, Number density of Cl^{-} in the left and right regions between the two particles.

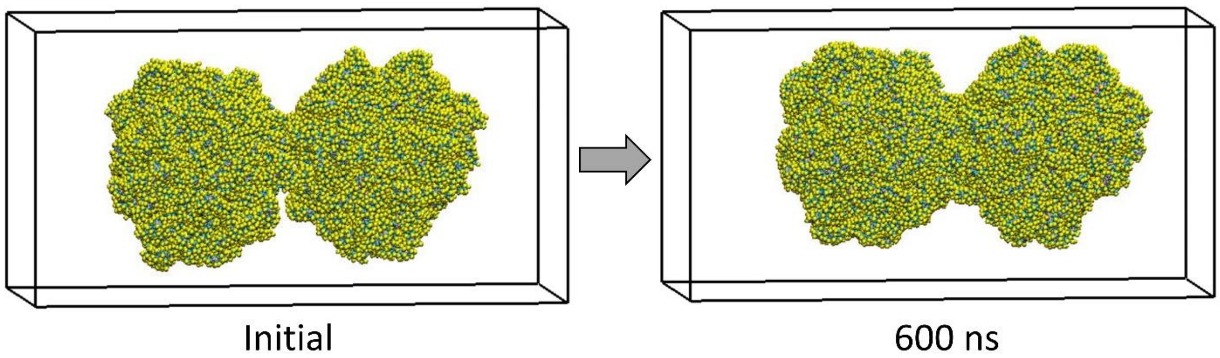

Supplementary Fig. 10. CGMD simulation in water when two particles are initially brought as close as possible. Yellow beads represent SPS. Na^{+}, Cl^{-}, and water molecules were omitted for clarity.

Supplementary Note 8: Bending experiment of colloidal chains.[21, 22]

The deflection y of a colloidal chain with a length L under an applied load F can be described by the Euler-Bernoulli beam equation, $y(x) = \frac{F}{6EI}(3Lx^2 - x^3)$, where E is the Young's modulus and I is the area moment of inertia. The bending rigidity of a single-bonded colloidal linear chain is expressed as $\kappa_{\text{chain}} = \frac{F_{\text{bend}}}{\delta}$. In Fig. 6f, the linear regression of the force profile resulted in $\kappa_{\text{chain}} \approx$ 0.41 pN·μm^{-1}. The single-bond rigidity κ_0 can be defined as $\kappa_0 = \kappa_{\text{chain}}\left(\frac{s}{R}\right)^3 = \frac{3\pi a_{\text{c}}^4 E_{\text{chain}}}{4R^3}$, where s is the chain contour length, R is the particle radius, E_{chain} is the Young's modulus of colloidal chain, and a_{c} is the radius of circular contact region between particles. For the colloidal chain composed of seven PS particles in Fig. 6d-f, κ_0 is found to be approximately 1.1 mN·m^{-1}. The a_{c} value can be estimated by the Johnson–Kendall–Roberts (JKR) theory for particle adhesion, given by $a_{\text{c}} = \left(\frac{3\pi R^2 W_{\text{SL}}}{2K}\right)^{\frac{1}{3}}$, where $K = \frac{2E_{\text{P}}}{3(1-\nu^2)}$ is the particle elastic modulus and W_{SL} is the adhesion energy between particles. Using the Young's modulus and the Poisson ratio of polystyrene, $E_{\text{P}} =$ 3.25 GPa and $\nu = 0.34$, respectively, the particle elastic modulus is estimated to be $K = 2.4$ GPa. At a diluted electrolyte condition, $W_{\text{SL}} = 93.9$ mN·m^{-1} can be obtained using the Young-Dupré equation $W_{\text{SL}} \approx W_{\text{SL}}^0 = \gamma_{\text{L}}(1 - \cos\theta_0)$, where the PS-water contact angle[23] is $\theta_0 = 73°$ and the water surface tension is $\gamma_{\text{L}} = 72.7$ mN·m^{-1}. Using the values of K and W_{SL}, $a_{\text{c}} = 73$ nm and $E_{\text{chain,PS}} = 0.05$ GPa are found. For example, the Young's moduli of polystyrene foam and low density polyethylene are ~0.005 and ~0.2 GPa, respectively.

Alternatively, the Young's modulus could be directly determined by fitting the colloidal chain profile with the Euler-Bernoulli beam equation. For instance, in the case of maximum bending (IV in Fig. 6e), its shape was fitted using the equation $y = \frac{F}{EI}\left(L\frac{(x-x_0)^2}{2} - \frac{(x-x_0)^3}{6}\right) + y_0$,

where $L = s$, which resulted in $\frac{F}{EI} = 0.0025 \pm 0.0003$ μm^{-2}. By substituting the value of F with $F_{\mathrm{bend}} = 1.78$ pN at $\delta = 4.25$ μm and using the relationship of $I = \frac{\pi a_{\mathrm{c}}^4}{4}$, the Young's modulus was $E_{\mathrm{chain,PS}} = 0.03$ GPa. This result aligns well with the previously obtained value of 0.05 GPa from the single-bond rigidity.

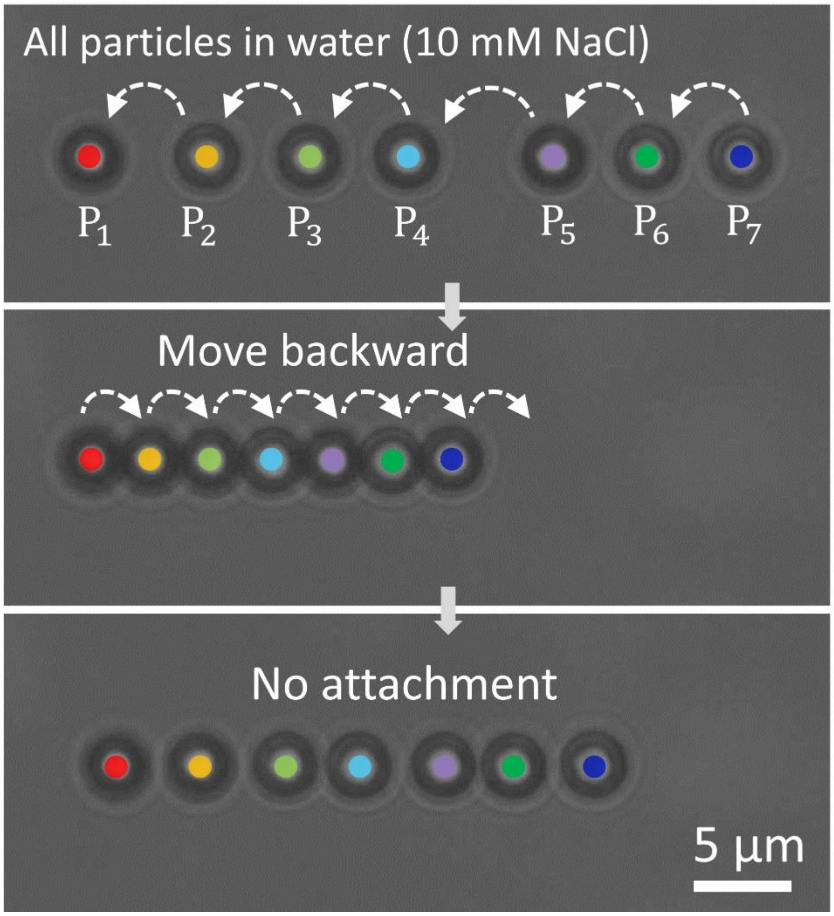

Supplementary Fig. 11. Attempt of a colloidal chain in 10 mM NaCl water. No colloidal chain was formed. The experiment was performed in the same manner as in Fig. 6c.

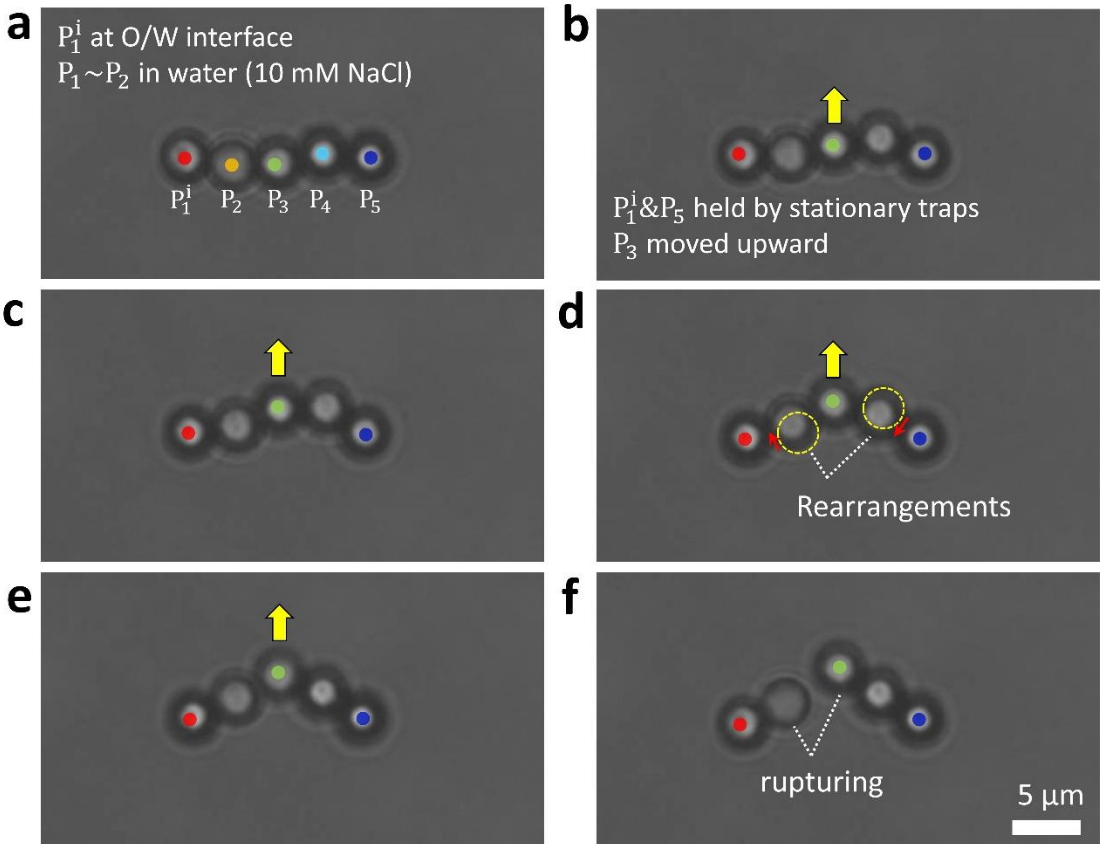

Supplementary Fig. 12. Micromechanics of a pentamer (Supplementary Movie 11). a, Initial configuration of a pentamer composed of one interfacial particle P_1^i and four water-immersed particles $P_2 - P_5$. All particles were optically trapped. **b,** Optical traps on P_2 and P_4 were removed. P_1^i and P_5 were fixed and P_3 was moved upward to apply the bending force to the pentamer. **c,d,** Small-scale rearrangements of P_2 and P_4 occurred along the directions indicated by the red arrows. The dotted yellow circles represent the positions right before the rearrangements. **e,f,** Rupture between P_2 and P_3 occurred when a critical bending moment was applied.

Supplementary Note 9: Formation of dimers in large-scale experiments.

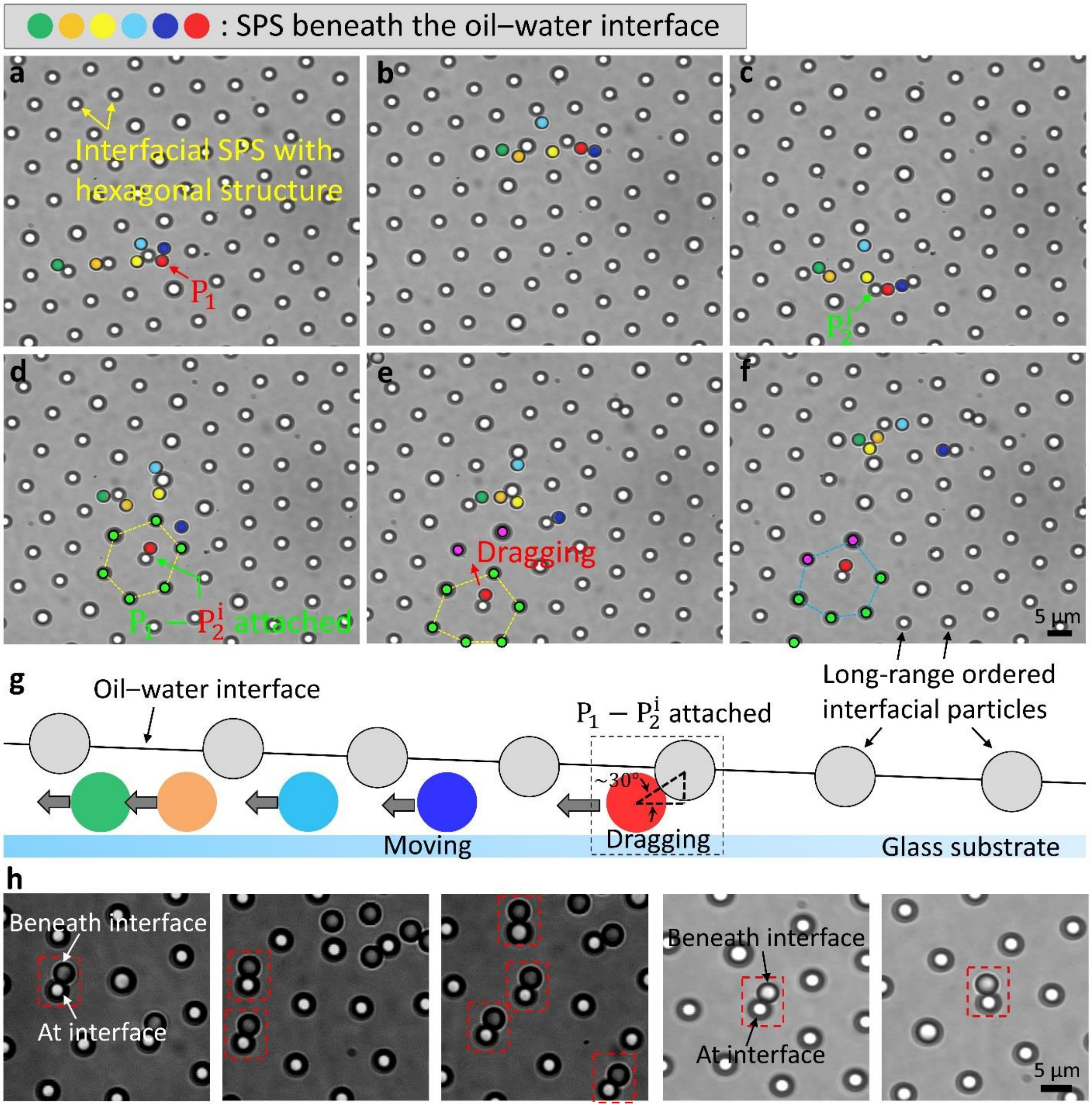

Supplementary Fig. 13. Formation of dimers in large-scale experiments. a-f, Selected images to show the dimer formation between a particle beneath the interface P_1 and a particle at the oil–water interface P_2^i (Supplementary Movie 12). The aqueous phase was pure water without any electrolytes. **g,** Schematic side view to illustrate the behavior shown in panels **a-f**. **h,** Other examples of the microscope images of dimer formation.

The SPS particles were spread at the oil-water interface and also dispersed in pure water. The interface height decreased by removing the subphase water using a micropipette until the interfacial particles and the particles between the interface and a glass substrate were simultaneously visible (Supplementary Fig. 13a). Notably, the brightness of each group looked different depending on the focal plane position due to the height difference in the two particle groups. In general, the interfacial particles appeared brighter than the water-immersed particles when the optical focus was at the interface. In addition, the interfacial particles could be easily distinguished in the optical microscope images because they showed a long-range ordered two-dimensional array due to the dipole-dipole repulsion between them,[13-15] and there were no such strong repulsions between the water-immersed particles beneath the interface (i.e., colored ones in Supplementary Fig. 13a). As shown in Supplementary Fig. 13a-f, the water-immersed particles moved toward the top-right, likely due to the interface slope.[19, 24] In this experimental condition, the water-immersed particles had chances to contact the surfaces of the interfacial particles that were exposed to water (Supplementary Fig. 13g) when they passed through the interfacial particles. At a certain moment when the P_1 beneath the interface met the P_2^i at the interface (Supplementary Fig. 13c), they formed a dimer and were captured in hexagonal cage particles indicated by the green dots (Supplementary Fig. 13d). The P_1 dragged the P_2^i along the direction of its original movement, but the long-range repulsions between the P_2^i and the interfacial cage particles prevented the movement. When the drag force caused by the P_1 overcame the repulsion-induced confinement, local rearrangement among interfacial particles occurred, and the dimer was confined in a new set of hexagonal cage particles (Supplementary Fig. 13e,f). Similar dragging and rearranging events of the interfacial particles were continuously observed. More examples of microscope images for dimer formation are displayed in Supplementary Fig. 13h. Considering the

three-phase contact angle ~99.4° of the interfacial P_2^i particle and the water-immersed P_1 particle that was almost in contact to the interface, the angle between the interface and the line joining the two particles was approximately $\sin\frac{1}{2} = 30°$. It was notable that the D–I interaction was strong enough to form the dimer when the two particles were not aligned orthogonally with respect to the interface.

Supplementary References

1. Kang D. W., Lim J. H. & Park B. J. Heterogeneous interface adsorption of colloidal particles. *Soft Matter* **13**, 6234-6242 (2017).
2. Kang D. W., et al. Geometric effects of colloidal particles on stochastic interface adsorption. *Langmuir* **34**, 8839-8847 (2018).
3. Pantina J. P. & Furst E. M. Directed assembly and rupture mechanics of colloidal aggregates. *Langmuir* **20**, 3940-3946 (2004).
4. Neuman K. C. & Block S. M. Optical trapping. *Review of scientific instruments* **75**, 2787-2809 (2004).
5. Park B. J. & Furst E. M. Optical trapping forces for colloids at the oil− water interface. *Langmuir* **24**, 13383-13392 (2008).
6. Ashkin A. Forces of a single-beam gradient laser trap on a dielectric sphere in the ray optics regime. *Biophys. J.* **61**, 569-582 (1992).
7. Park B. J. & Furst E. M. Effects of coating on the optical trapping efficiency of microspheres via geometrical optics approximation. *Langmuir* **30**, 11055-11061 (2014).
8. Schneider C. A., Rasband W. S. & Eliceiri K. W. NIH Image to ImageJ: 25 years of image analysis. *Nat. Methods* **9**, 671-675 (2012).
9. Crocker J. C. & Grier D. G. Methods of digital video microscopy for colloidal studies. *J. Colloid Interface Sci.* **179**, 298-310 (1996).
10. https://site.physics.georgetown.edu/matlab/.
11. Atkins P., Atkins P. W. & de Paula J. *Atkins' Physical Chemistry*, 10th edn. Oxford Univ. Press (2014).
12. Israelachvili J. N. *Intermolecular and surface forces*, 2nd edn. Academic Press (1991).
13. Choi K. H., et al. Interpretation of Electrostatic Self-Potential Measurements Using Interface-Trapped Microspheres with Surface Heterogeneity. *ACS Appl. Polym. Mater.* **2**, 1304-1311 (2020).
14. Park B. J., et al. Direct measurements of the effects of salt and surfactant on interaction forces between colloidal particles at water-oil interfaces. *Langmuir* **24**, 1686-1694 (2008).
15. Hurd A. J. The electrostatic interaction between interfacial colloidal particles. *J. Phys. A* **18**, L1055 (1985).
16. Oettel M. & Dietrich S. Colloidal interactions at fluid interfaces. *Langmuir* **24**, 1425-1441 (2008).
17. Hiemenz P. C. & Rajagopalan R. *Principles of colloid and surface chemistry*, 3rd edn. Marcel Dekker (1997).
18. Paunov V. N. & Binks B. P. Analytical expression for the electrostatic disjoining pressure taking into account the excluded volume of the hydrated ions between charged interfaces in electrolyte. *Langmuir* **15**, 2015-2021 (1999).
19. Park B. J., Lee M., Lee B. & Furst E. M. Lateral capillary interactions between colloids beneath an oil–water interface that are driven by out-of-plane electrostatic double-layer interactions. *Soft Matter* **11**, 8701-8706 (2015).
20. Biriukov D., Fibich P. & Předota M. Zeta potential determination from molecular simulations. *J. Phys. Chem. C* **124**, 3159-3170 (2020).
21. Pantina J. P. & Furst E. M. Elasticity and critical bending moment of model colloidal aggregates. *Phys. Rev. Lett.* **94**, 138301 (2005).

22. Pantina J. P. & Furst E. M. Micromechanics and contact forces of colloidal aggregates in the presence of surfactants. *Langmuir* **24**, 1141-1146 (2008).
23. Paunov V. N. Novel method for determining the three-phase contact angle of colloid particles adsorbed at air− water and oil− water interfaces. *Langmuir* **19**, 7970-7976 (2003).
24. Park B. J. & Furst E. M. Fluid-interface templating of two-dimensional colloidal crystals. *Soft Matter* **6**, 485-488 (2010).
