# Supplementary material for: Direct measurements of the colloidal Debye force
Source: Nat Commun. 2023 Jun 29;14:3838. doi: 10.1038/s41467-023-39561-8 (PMC10307906; doi:10.1038/s41467-023-39561-8)
Supplement: Supplementary file 3 — Description of Additional Supplementary Files [file 41467_2023_39561_MOESM3_ESM.pdf]

Description of Additional Supplementary Files

Supplementary Movie 1. Attempt to attach a water-immersed particle to the oil–water interface using optical laser tweezers. The particle was attached to the interface. The water phase contained 10 mM NaCl.

Supplementary Movie 2. Attempt to attach a water-immersed particle to the oil–water interface using optical laser tweezers. The particle was not attached to the interface. The water phase contained 10 mM NaCl.

Supplementary Movie 3. Attempt to form a $P_1^i - P_2$ dimer using optical laser tweezers. The dimer was formed. The water phase contained 10 mM NaCl.

Supplementary Movie 4. Attempt to form a $P_1^i - P_2$ dimer using optical laser tweezers. The dimer was not formed. The water phase contained 10 mM NaCl.

Supplementary Movie 5. Attempt to form a $P_1^i - P_2 - P_3$ trimer using optical laser tweezers. The trimer was formed. The water phase contained 10 mM NaCl.

Supplementary Movie 6. Optical trap on P_2 of the $P_1^i - P_2 - P_3$ trimer was removed. The P_2 position was maintained. The water phase contained 10 mM NaCl.

Supplementary Movie 7. Optical trap on P_3 of the $P_1^i - P_2 - P_3$ trimer was removed. The P_3 position was maintained. The water phase contained 10 mM NaCl.

Supplementary Movie 8. Attempt to form a $P_1 - P_2 - P_3$ trimer in water using optical laser tweezers. The trimer was not formed. P_2 disappeared when the optical trap on P_2 of the $P_1 - P_2 - P_3$ trimer was removed. The water phase contained 10 mM NaCl.

Supplementary Movie 9. Formation of a heptamer. The water phase contained 10 mM NaCl.

Supplementary Movie 10. Bending micromechanics of a heptamer. The water phase contained 10 mM NaCl.

Supplementary Movie 11. Bending micromechanics of a pentamer. The water phase contained 10 mM NaCl.

Supplementary Movie 12. Formation of dimers in large-scale experiments. Pure water was used.
